# Habitat heterogeneity and green filamentous algae influence the larval ecology of *Anopheles stephensi* during the dry season in Eastern Ethiopia

**DOI:** 10.1186/s13071-025-07100-7

**Published:** 2025-11-14

**Authors:** Araya Gebresilassie, Esayas Aklilu, Solomon Yared, Elyas Abdulahi, Kedir Adem Darasa, Ahmed Ali habib, Witka nore Witka, Hamedu Ahmed, Dagnew Hagezom, Tamiru Kassa, Dorian Jackson, Gonzalo M. Vazquez-Prokopec

**Affiliations:** 1 Department of Zoological Sciences, Addis Ababa University, Addis Ababa, Ethiopia; 2 Aklilu Lemma Institute of Health Research, Addis Ababa University, Addis Ababa, Ethiopia; 3 Department of Biology, Jigjiga University, Jijiga, Ethiopia; 4 Department of Geography Population, Resources and Environmental Economics, Jigjiga University, Jigjiga, Ethiopia; 5 Afar Regional Health Bureau, Semera, Ethiopia; 6 Afar Public Health and Research Institute, Semera, Ethiopia; 7 Emory University Ethiopia Office, Addis Ababa, Ethiopia; 8 Department of Environmental Sciences, Emory University, 400 Dowman Dr, Atlanta, GA 30322 USA

**Keywords:** *Anopheles stephensi*, Larval ecology, Urban malaria, Dry season, Ethiopia, Habitat productivity, Larval source management, Lefkovitch matrix model

## Abstract

**Background:**

The recent invasion of *Anopheles stephensi*, an urban-adapted malaria vector, poses a threat to malaria elimination efforts in Africa. Understanding the larval ecology of this mosquito during the dry season, which represents a potential population bottleneck due to limited larval habitats and harsher environmental conditions, is critical for informing targeted interventions.

**Methods:**

We conducted systematic surveys in three climatically distinct Ethiopian cities—Semera, Logiya and Jigjiga—during the dry season of 2023. A total of 523 water-holding habitats were identified and characterized for the presence and productivity of *An. stephensi* immature stages. Habitat characteristics, including container type, floating algal mass presence, cover status and water chemistry, were recorded. A Lefkovitch matrix model was used to project habitat-specific productivity.

**Results:**

Overall, 40.9% of the habitats surveyed were positive for *An. stephensi*. Larval positivity and productivity were significantly higher in Semera and Logiya, coinciding with warmer temperatures and lower elevations. Three habitat types, namely construction pits, residential cisterns and ground-level water tanks, accounted for 87% of positive habitats and 81% of all larvae. Complete stage structures observed in key habitats indicated ongoing oviposition and larval development throughout the dry season. The Lefkovitch model identified construction pits as the most productive habitat type across all three cities. *Anopheles stephensi* larval presence and density were strongly associated with the presence of green filamentous algal aggregates (odds ratio [OR] 6.00, 95% confidence interval [CI] 2.76–13.04). Secondary predictors were lack of cover (OR 0.98, 95% CI 0.96–0.98) and specific water chemistry parameters (OR 1.21, 95% CI 1.03–1.42).

**Conclusions:**

Urban infrastructure and water storage practices during the dry season support discrete ecological niches that facilitate the persistence of *An. stephensi* populations in eastern Ethiopia. These findings underscore the importance of targeted larval source management focused on key habitat types, particularly construction-related and domestic water storage containers, to reduce urban malaria transmission risk in the Horn of Africa.

**Graphical abstract:**

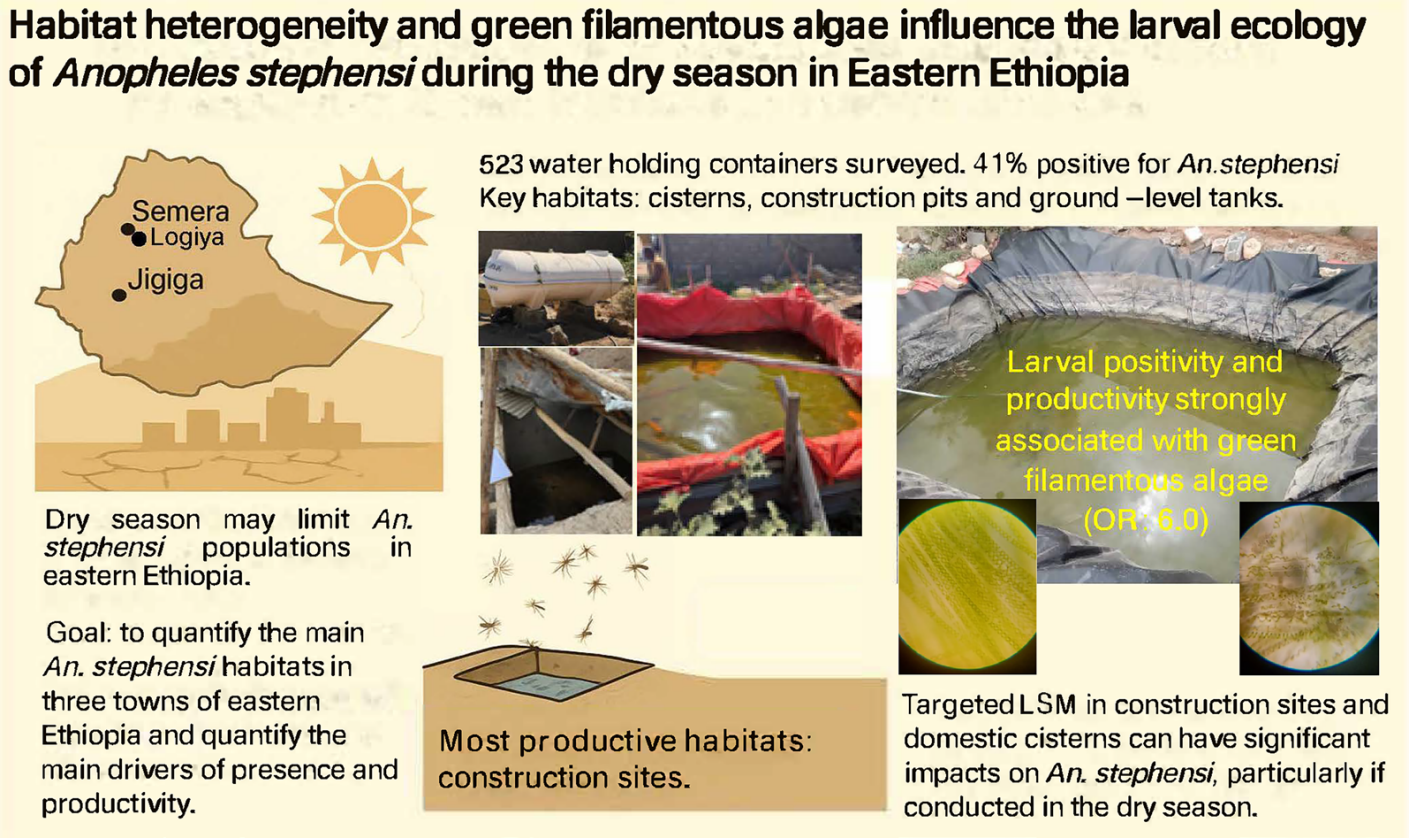

**Supplementary Information:**

The online version contains supplementary material available at 10.1186/s13071-025-07100-7.

## Background

*Anopheles stephensi* is a highly efficient malaria vector native to India, Pakistan and parts of the Arabian Peninsula, where it is known to transmit both *Plasmodium falciparum* and *Plasmodium vivax *[1]. Since its initial detection in Djibouti in 2012 [2], *An. stephensi* has rapidly expanded across the African continent, with confirmed reports in Ethiopia [3], Sudan [4], Somalia [5], Kenya [6], Eritrea [7], Nigeria [7] and Ghana [8]. This expansion poses a significant threat to malaria control and elimination efforts across sub-Saharan Africa, particularly in urban and peri-urban settings [1, 9].

The establishment of *An. stephensi* in the Horn of Africa has already been linked to alarming increases in urban malaria incidence. In Djibouti, for example, malaria cases rose dramatically from 2.5 per 1000 people in 2013 to 97.6 per 1000 in 2020 following the species’ introduction [10]. Similarly, clusters of human* P. vivax*- and* P. falciparum*-associated illnesses in the urban center of Dire Dawa, Ethiopia, were statistically linked to the finding of *An. stephensi*-positive larval habitats nearby [11]. In other Ethiopian towns, recent increases in malaria case incidence can possibly also be linked to *An. stephensi* invasion [12]. These cases underscore the species’ potential to establish sustained transmission in previously low-risk, urban or semi-arid areas.

Although a number of studies have documented the presence, distribution and insecticide resistance profiles of *An. stephensi* in Ethiopia [13–15], significant gaps remain regarding its larval ecology and spatial distribution across varied urban landscapes. This vector is known to exploit a wide range of artificial and natural aquatic habitats, including water storage containers, discarded tires, construction sites and cisterns [14, 15]. A recent meta-analysis comparing the bionomics of *An. stephensi* across its native and invasive ranges revealed that while the species exploits a wide variety of natural and artificial habitats in its native range, in invaded areas it relies disproportionately on man-made containers [16]. In India, the presence of different *An. stephensi* ‘forms’ (described based on egg morphology but indistinguishable genetically) that have different habitats and a predominant rural and zoophilic “mysorensis” form may help explain the plasticity in habitat use and ecology in its native range [1, 17]. Information from two studies suggest that the extended dry season of Ethiopia may function as a significant population bottleneck for *An. stephensi* by limiting the type and number of suitable habitats as well as larval habitat conditions [9, 18]. As the dry season in eastern Ethiopia progresses, *An. Stephensi* productivity significantly concentrates in large water reservoirs (for drinking and construction) that not only are used predominantly for residential or construction purposes but also became the refugia of the population during the harsh dry season [18]. Of particular relevance to this seasonal persistence are the changes in the physical, chemical and biological conditions of potential larval habitats that may influence mosquito productivity and population dynamics.

Understanding the larval ecology of *An. stephensi* in the African context is essential for the development of effective control strategies. Larval source management (LSM), which targets mosquito larvae in their aquatic habitats, is especially viable in these settings where breeding sites are relatively few, fixed, and identifiable [19]. However, the successful application of LSM relies on detailed knowledge of the ecological features that favor larval development and population persistence, including water source type, vegetation, algae presence, shading and human water storage practices [19]. In Ethiopia, construction sites and household water storage during the dry season likely play a key role in sustaining *An. stephensi* populations [9, 18]. Understanding and characterizing these habitats and their contribution to vector productivity is therefore essential for informing targeted vector control.

The aim of this study was to examine the spatial distribution and ecological characteristics of *An. stephensi* larval habitats in the northeastern and eastern regions of Ethiopia. By identifying key factors associated with larval presence and developmental stage, this work provides essential insights to inform the design and implementation of effective, context-specific larval control strategies.

## Methods

### Study design

**Fig. 1:**
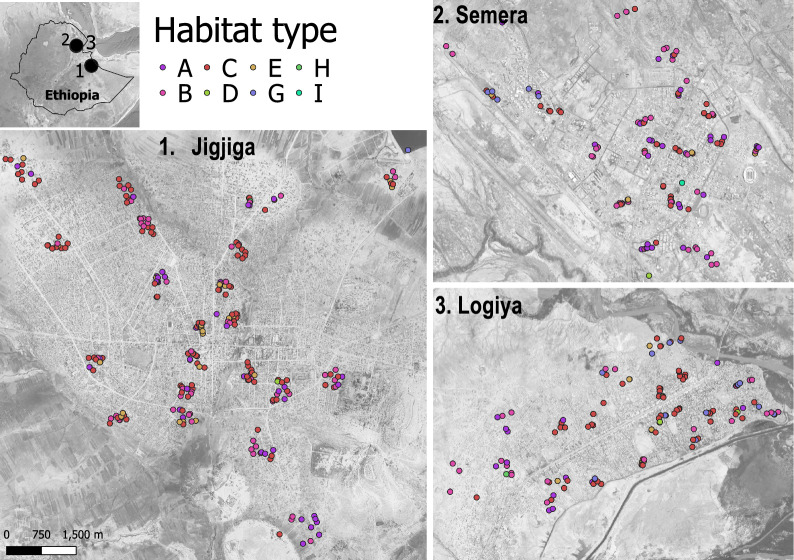
Map of the three study urban centers and distribution of habitat types. Location of all habitats sampled in the Jigjiga, Semera and Logiya towns located in eastern Ethiopia during the dry season of 2023, separated by mosquito habitat type, indicated by color coding. Code for habitat type:* A* Residential cistern,* B* construction pits,* C* ground-level tanks,* D* tires,* E* small plastic containers,* F* bottles,* G* puddles from rain or burst pipes,* H* car wash drainage,* I* surface water. See Additional file 2: Figure S2 for details of each habitat. All maps are shown on the same scale

This study was conducted in three Ethiopian urban centers: Semera, Logiya and Jigjiga (Fig. 1). All three of these towns are experiencing rapid growth due to political stability and economic opportunities. Semera (11°7′94″N, 41°0′08″E; elevation 433 m a.s.l.; 2023 population 6784) is the capital of the Afar region, and is located 585 km northeast of Addis Ababa along the Addis Ababa-Djibouti highway, an important trade corridor. Logiya (11°43′19″N, 40°58′28″E, elevation 393 m a.s.l.; 2023 population 36,313) is located 7 km north of Semera. Both towns have unimodal and erratic rainfall patterns and receive an annual average rainfall of 358 mmm concentrated between June and September. At an average annual temperature of 32.3 °C, both towns experience extreme heat, with June being the hottest month (average maximum temperatures reaching 44 °C); January is the coldest month (24.2 °C). Jigjiga (9°36′N, 42°48′E; elevation 1634 m a.s.l.; 2023 population 800,000) is the capital city of the regional Somali state, eastern Ethiopia (Fig. 1). Jigjiga is located 615 km from Addis Ababa on the Addis Ababa-Port Berbera highway, the second largest trade route in Ethiopia. The average annual rainfall in Jigjiga is about 600 mm, characterized by bi-modal seasonality (main rainfall in July–September, followed by short rainfall April–June). Jigjiga experiences a mean annual temperature of 20 °C. Malaria does not occur locally in Jigjiga, but seasonal malaria transmission occurs in lowland rural areas located approximately 25 km from the city.

Multiple factors were taken into consideration for the selection of the three urban centers. Jigjiga was selected due to its unique situation of being invaded by *Anopheles stephensi* but having a history of local malaria transmission. Given the lack of *Anopheles gambiae* sensu lato (*An. gambiae* s.l.) in or around the town, the selection of Jigjiga was based on the establishment of a long-term study focused on towns recently invaded by *An. stephensi* that may experience local malaria transmission as the vector establishes. Semera and Logiya were selected due to their closeness to Djibouti and a history of local malaria transmission in riverine communities by *Anopheles arabiensis* as well as the recent increase in malaria cases potentially attributed to *An. stephensi* [12].

The study design was centered on the urban–rural gradient study design commonly used in urban ecology studies [9, 20]. Briefly, centering in the historical ‘downtown’ of a town (identified using satellite imagery), four sectors according to the cardinal points (i.e. NW, NE, SW and SE) were drawn on a map using natural divisions such as main roads or rivers whenever possible (Additional file 1: Figure S1). Within each sector, five sampling clusters (i.e. conglomerates of 4 × 4 or 5 × 5 city blocks; Additional file 1: Figure S1) selected across the urban–rural footprint of each cardinal point were identified. This design was suitable to characterize *An. stephensi* distribution and ecology because it allowed larval habitat productivity to be characterized throughout habitats that spanned the entire urban footprint, rather across randomly selected houses. In addition, by sampling across the urban footprint of each town, we ensured findings are representative of different socio-economic, ecological and housing conditions (from dispersed semi-rural traditional houses to highly urbanized housing units).

Sampling clusters consisted of two to four city blocks. For each cluster in Semera and Logiya, two construction sites and five residential premises—for a total of 140 premises per town—were surveyed; in Jigjiga 10 premises in each sampling cluster (including 2 construction sites) were selected—for a total of 220 premises (Fig. 1). In cases where construction sites were absent, two additional residential premises were included in the sampling cluster. The study was conducted from 7 March to 26 March 2024 in Semera and Logiya towns and from 8 March to 28 March in Jigjiga. These periods were the peak of the dry season in the three study sites (Additional file 1: Figure S1). Given most small habitats often infested by *An. stephensi* are expected to dry during this period [18], sampling during the dry season provided an opportunity to study key habitats for population persistence.

### Mosquito larval and pupal sampling

Mosquito larvae and pupae were sampled from each water-holding container/habitat found within the premises of each house and its immediate perimeter using a standard mosquito dipper (350 ml) [9]. Number of dips was a function of container/habitat size: large containers (residential cisterns, construction pits) were dipped 20 times, medium-sized containers (200-l drums) were dipped 10 times and smaller containers (tires, buckets) were exhaustively examined and all larvae and pupae collected. The distribution of dips per habitat aimed at covering the entire surface, aiming at corners as well as floating vegetation/debris where immature mosquitoes may be hiding. The coordinates of each water holding container/ habitat was recorded using a hand-held global positioning system (GPS) (Garmin GPS 60; Garmin International, Olathe, KS, USA). The sampled larvae and pupae from each habitat were placed in an 18-oz. sterile plastic bag (Whirl–Pak; Filtration Group, Austin, TX, USA) containing water from the same habitat (to minimize stress and mortality) and transported to the laboratory where the numbers of first and second instar larvae, third and fourth instar larvae and pupae were recorded, differentiated by species. All larvae were allowed to pupate by feeding yeast powder. The pupae were transferred from the enamel trays to small beakers containing a small amount of water and put inside a bugdorm (30 × 30 × 30 cm) for emergence. Adult *Anopheles* mosquitoes that emerged from collected larvae and pupae were identified using a morphological identification key. Molecular confirmation of *An. stephensi* was conducted on a subset of 200 larvae chosen at random and kept in DNA Shield stabilization solution (Zymo Research, Irvine, CA, USA) using standard primers and protocols [3].

### Characterization of larval habitats

The habitat characteristics of any water-holding habitat that tested either positive or negative for mosquito larvae or pupae encountered during sampling were measured. The physical characteristics of breeding habitats were documented, including habitat type, material (plastic, cement, metal), location, dimensions, water source (tap, rain, river) and environmental factors (e.g. shade, cover, algae presence). Given the high variability of habitat types, a number of broad categories were identified and used to group observations. Habitat type was thus defined by the following classes (Additional file 2: Figure S2): A = residential cistern, B = construction pits, C = ground level tanks, D = tires, E = small plastic containers, F = bottles, G = puddles from rain or burst pipes, H = car wash drainage and I = surface water. A residential cistern was generally built of concrete, measured approximately 3 × 3 m in surface area and had variable depth (from a few centimeters to meters). The main feature of this type of habitat was that water was used for human consumption. Construction pits could be made of either plastic sheets buried in the ground and elevated by dirt edge or a wood and metal frame, or made of cement (e.g. residential cisterns). The main feature was that their purpose and water were for supporting construction activities (either water for mortar cement, brick manufacturing or other activities not related to residential water consumption). Ground-level tanks were typically made of plastic, and less commonly of metal, and were of various sizes and capacities. The water stored in these tanks was primarily used for human or animal consumption. Tires were either individual or grouped in piles and had different sizes, with car and truck tires being the most common. The category of small plastic containers included buckets, plastic jars and small containers that were primarily rain filled. Bottles were primarily rain filled and varied in volume from 350 ml to 2 l. In Semera-Logiya, we found puddles in the street that were persistent in the dry season; these were generated by constant flow of water from burst pipes. Given this unique nature and source of water, we included these puddles as a category independent from surface water, which alludes to natural water habitats (river, pond, rain-filled reservoirs). Water drainage occurred on properties/areas where motorcycles, cars and trucks were washed, with water often accumulating to form puddles (Additional file 2: Figure S2).

The amount of shade at each habitat was quantified as a binary variable, and visually assessed by the field team. Shade was present when there was clear evidence that the habitat was not fully exposed to the sun, with shade outdoors provided, for example, by trees or roofs. Any indoor container was considered to be shaded. Habitat cover was visually assessed based on the amount of physical cover each habitat had. The variable was based on percentiles (0 = no cover, up to 25%, 50%, 75% cover and fully covered). Algae presence was specifically assessed and quantified as the presence of filamentous algal aggregates, as preliminary work by our team identified filamentous algal aggregates as microenvironments where often *An. stephensi* larvae are found. These algal aggregates were also described by color (green or other color [e.g. red/orange or gray]). Water chemistry parameters (pH, temperature, salinity, total dissolved solids [TDS], conductivity) were recorded using a digital meter (model WQ120; Triplett Test Equipment, Manchester, NH, USA). Habitat dimensions and water depth were measured with a tape. All data collected in the field were electronically entered into tablet-based formats in KoboToolbox (https://www.kobotoolbox.org).

### Statistical analyses

We focused on two measures to characterize the larval ecology of *An. stephensi*. Habitat positivity was quantified as the proportion of habitats with *An. stephensi* present, divided by all habitats with water that were surveyed. Habitat productivity was characterized by the count of all *An. stephensi* found within each habitat. For productivity, we further divided the counts to characterize the stage structure of *An. stephensi* by habitat by aggregating immature stages in young (I-II instar), old (III-IV instar) and pupae. All measures of habitat positivity were statistically analyzed using binomial generalized linear mixed models (GLMM) using sampling cluster ID as the random intercept. The justification of using a GLMM emerged from the fact that the data was hierarchical in nature (random houses were sampled from multiple clusters, which were located in different sectors of each town). To account for potential differences from being in different areas of each town, we included a random effect for the ID of each cluster, as explained in the following text.

Residential cisterns (a very common and widely used habitat in Ethiopia) were used as a baseline for statistical comparisons. For productivity, negative binomial GLMMs were implemented, similarly using sampling cluster ID as the random intercept. Models for positivity and productivity had the following structure (with data format shown in parenthesis):

$$\left[An. stephensi\right] \sim {Cover}_{(0-100\%)}+{Algae}_{(\text{0,1})}+{Habitat}_{(A vs C-H)}+{Turbidity}_{(turbid, medium, clean)}+{City}_{(Semera, Logia,Jigjiga)}+{Water quality}_{(PCA1)}+\left(1 \right|Cluster ID)$$

All analyses were run in R 4.3.1, using package *lme4* [21]. Prior to running the GLMMs, we checked all the variables for correlation using correlation matrices. The finding of a strong correlation between salinity, conductivity and TDS but not of pH prompted the application of principal component analysis (PCA) to generate a “water quality index” that encapsulated all. The first component (PC1) was then used in all models as a measure of water quality.

We characterized mosquito population structure by counting the number of young larvae (I–II instar), old larvae (III–IV instar) and pupae per habitat. Instead of using standardized measure (number per dip), we used the total catch as a measure of the absolute contribution of each habitat. This number was used to characterize habitat types in their level of completeness of population structure (e.g. the finding of a complete stage structure is indicative of multiple oviposition events leading to overlapping generations). To better exemplify the potential contribution to the adult population by habitat type, we used the empirical stage distribution for each habitat and town as initial conditions to multiply the Lefkovitch transition matrix [22] shown below:

$$L=\left(\begin{array}{cccc}0& 0& 0& 0\\ s1& 0& 0& 0\\ 0& s2& 0& 0\\ 0& 0& s3& 0\end{array}\right)$$,

with parameters representing the survival probability of young instars into old instars (*s*1), of old instars into pupae (*s*2) and adult emergence rate (*s*3). The matrix excludes values on the first row, given we were not interested in modeling recruitment into each habitat. We considered differential survival by instar, with *s*1 = 0.50, *s*2 = 0.75, *s*3 = 0.85. Such values were based on expert opinion, as life table estimates of *An. stephensi* in Africa are lacking. The value of *s*1, *s*2 and *s*3 was kept the same for each habitat type and city. These parameters were chosen to characterize a possible transition, and further research should focus on proper estimates for better model parameterization.

## Results

### Larval positivity

**Fig. 2:**
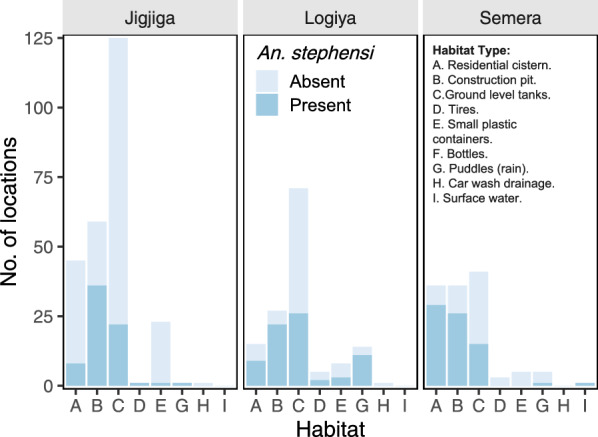
*Anopheles stephensi* habitat positivity by study town and habitat type. Positivity was measured as the number of locations where *An. stephensi* was collected. See Additional file 2: Figure S2 for details of each habitat. Category F (bottles) was sampled but no mosquitoes were found across all towns and this habitat category was therefore eliminated from the figure to improve clarity

The survey of a total 438 premises across the three towns (107 premises in Semera, 112 in Logiya and 219 in Jigjiga) led to the finding of 523 unique water-holding containers (127 in Semera, 141 in Logiya and 255 in Jigjiga). Overall, a total of 214 (40.9%) water-holding containers were found to be positive for *An. stephensi* presence (Fig. 2). Other species found included *Aedes aegypti* (5.7%), *Culex quinquefasciatus* (14.9%), *Anopheles gambiae* s.l. (1.9%) and *Culiseta* spp. (0.2%). When broken down by town, *An. stephensi* larval habitat positivity was significantly higher in Semera (51.8%; odds ratio [OR] 2.89, 95% confidence interval [CI] 1.88–4.45; *P* < 0.01) and Logiya (56.7%; OR 3.53, 95% CI 2.26–5.52; *P* < 0.01) compared to Jigjiga (27.1%).

All sites sampled were grouped within nine categories to quantify *An. stephensi* larval positivity by habitat type (Fig. 2). Three habitat categories (residential cisterns, construction pits and ground-level tanks) were the most common (455 of 523 sampled sites; 87%) across all three towns, and also the most frequently infested with *An. stephensi* (Fig. 2). In Semera and Logiya, *An. stephensi* was found infesting a few (*N* = 20) puddles generated by the burst of water supply pipes (Fig. 2). Additional file 2: Table S1 provides full pairwise comparisons among habitat types. When positivity between habitats was statistically compared, using residential cisterns as the baseline, construction pits were the only habitats significantly more positive, whereas ground-level tanks and small plastic containers were significantly less positive (Additional file 2: Table S1; Fig. 2).

### Larval productivity

In total, 9160 immature *An. stephensi*, 363 *Ae. aegypti*, 533 *Cx. quinquefasciatus*, 24 *An. gambiae* s.l. and seven *Culiseta* spp. were collected from the three towns. PCR confirmed the identity of a subset of 200 larvae that were visually identified as *An. stephensi*. Despite having more habitats sampled, Jigjiga only accounted for 900 (9.8%) of all *An. stephensi* immatures (Table 1). When comparing towns, this difference in immature collection was significantly different, with a significantly higher collection of immatures in Logiya and Semera than in Jigjiga (Additional file 2: Table S2). In terms of the number of immatures per dip, there was a greater contrast between the three towns than in the total count per habitat (Additional file 2: Table S2). As observed with *An. stephensi* positivity, Semera and Logiya did not differ significantly regarding their productivity (Additional file 2: Table S2).

**Table 1:** Number of immature *Anopheles stephensi* collected across all habitat types in the three urban centers included in the study

Urban center (town)	Metric	Habitat type									Total no. collected across all habitat types
		A: Residential cistern	B: Construction pits	C: Ground-level tanks	D: Tires	E: Small plastic containers	F: Bottles	G: Puddles from rain or burst pipes	H: Car wash drainage	I: Surface water	
Jigjiga	Total surveyed (*n*)	45	59	125	1	23	1	1	1		256
	Total larvae (*n*)	60	418	244	1	2	0	8	0	0	733
	% larvae	8%	57%	33%	0%	0%	0%	1%	0%	0%	100%
	Total pupae (*n*)	16	99	51	0	0	0	1	0	0	167
	% pupae	10%	59%	31%	0%	0%	0%	1%	0%	0%	100%
Logiya	Total surveyed (*n*)	15	27	71	5	8	0	14	1	0	141
	Total larvae (*n*)	328	2003	1404	88	4	0	747	0		4574
	% larvae	7%	44%	31%	2%	0%	0%	16%	0%	0%	100%
	Total pupae (*n*)	30	49	105	19	0	0	41	0	0	244
	% pupae	12%	20%	43%	8%	0%	0%	17%	0%	0%	100%
Semera	Total surveyed (*n*)	36	36	41	3	5	3	5		1	130
	Total larvae (*n*)	1255	1746	242	0	4	0%	15		1	3263
	% larvae	0.384615	0.53509041	0.07416	0	0.001226	0	0.004597	0	0.0003	1
	Total pupae (*n*)	45	112	12	0	0	0%	0		10	179
	% pupae	0.251397	0.62569832	0.06704	0	0	0	0	0	0.0559	1

**Fig. 3:**
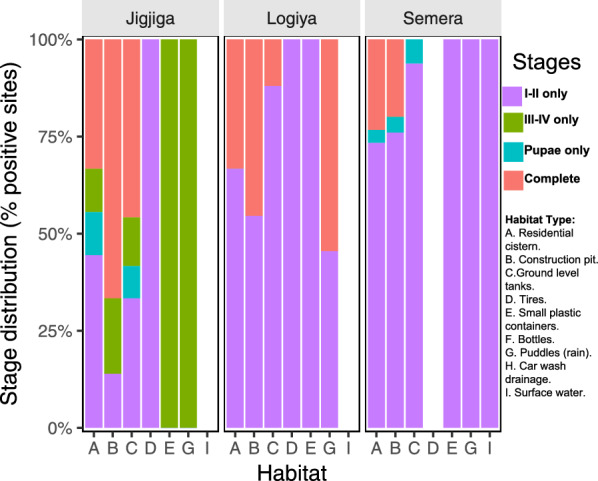
*Anopheles stephensi* stage structure by town and habitat type., Stage structure was measured as the percentage of total mosquito immatures collected by habitat and town. Stages were differentiated as young (I–II instar larvae), old (III–IV instar larvae) or pupae. A habitat was considered to have a complete stage structure when at least one immature of each class was found in that habitat (labeled as “Complete” in figure). Letters indicate the habitat type (defined to the right of figure). See Additional file 2: Figure S2 for details of each habitat

Across the three study sites, and similarly to positivity, presence of immatures collected was concentrated in three major larval habitats: residential cisterns, construction pits and ground-level tanks (Table 1). Across all towns, 19% of sampling sites (*n* = 41) contributed 81% of all larvae. Of these 41 highly productive habitats, 13 (31.7%) were residential cisterns, 15 were construction pits (36.6%), eight (19.5%) were ground-level barrels, four (9.8%) were puddles and one (2.4%) was a tire. While total mosquito counts can be used as a biomarker to identify habitats and their potential contribution to population recruitment and growth, the quantification of the stage structure of a population in a given habitat is a more accurate estimate of the role of each habitat in contributing to population recruitment and growth. In the absence of rainfall for almost 2 months prior to the survey, the finding of a complete stage structure can be considered a sign of recurrent cycles of oviposition and adult emergence, driven by permanence of water in the habitat. However, the finding of a young population (larval stages I–II only) may indicate recent oviposition in a habitat that has not been colonized previously or one that has been just recently artificially filled with water. Across the three study sites, residential cisterns, construction pits and ground-level tanks were the habitats with the highest proportion of complete stage distributions (Fig. 3). In Jigjiga, construction pits dominated not only in the number of immatures collected but also in the proportion of habitats with complete stage distributions (60%; Table 1; Fig. 3). Semera and Logiya showed a similar but less marked trend (20% and 45%, respectively), with ground-level tanks playing a less important role (13%; Fig. 3). Interestingly, tires were only colonized by young *An. stephensi* instars, suggesting a limited contribution to productivity (Fig. 3). Additionally, while ground puddles showed a high potential for productivity in Logiya (Fig. 3), the low numbers detected (Fig. 1) reduce their potential absolute contribution.

**Fig. 4:**
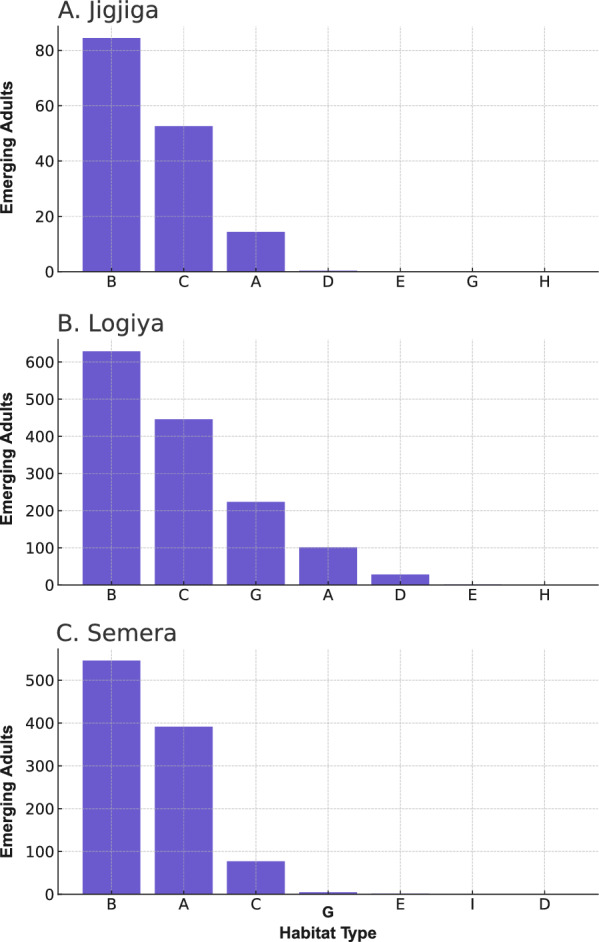
Number of adult* Anopheles stephensi* predicted to emerge from each habitat type. The values of adults were estimated by multiplying the empirical stage distribution to the Lefkovitch transition matrix, *L*, calculated for each town. The result is the total number of adults after three cycles of the matrix. Habitats are ranked by their contribution to the total adult population. Letters indicate the habitat type:* A* Residential cistern,* B* construction pits,* C* ground-level tanks,* D* tires,* E* small plastic containers,* F* bottles,* G* puddles from rain or burst pipes,* H* car wash drainage,* I* surface water. See Additional file 2: Figure S2 for details of each habitat. The* Y*-axis is scaled to the maximum productivity quantified for each town

Using the observed stage structure by habitat type and urban center (Additional file 2: Table S3) and the transition matrix *L*, we estimated the projected number of adults emerging from each habitat, assuming no differential mortality by habitat type (Fig. 4). After three cycles, all larvae and pupae had either died or transitioned to adult mosquitoes. The predicted number of adults emerging per habitat type supported the importance of construction pits as productive habitats for *An. stephensi* across all study sites. When the frequency, productivity and stage structure were considered together, ground-level tanks were predicted to be the second most productive habitats in Jigjiga and Logiya but not in Semera, where residential cisterns were the second most important contributor to adult mosquitoes (Fig. 4). Another interesting finding was that puddles generated by damaged water lines were the third most important contributor to productivity in Logiya (Fig. 4), although their high contribution was the result of a single puddle having > 200 *An. stephensi* in it.

### Factors explaining *An. stephensi* positivity and productivity in key habitats

As our results showed that most of the differences in positivity and productivity were explained by habitat type, we then analyzed *An. stephensi* data from the three main three habitats (A, B, C), which accounted for most of the positivity and immatures, to understand what the reasons for this variability. Before running the models, we assessed correlation among predictor variables and found that most water chemistry metrics were highly correlated (Additional file 2: Figure S3). To avoid model overfitting, we combined them into a PCA and used PC1 as a predictor variable for “water chemistry” (Additional file 2: Figure S4). PC1 explained 69.2% of the variance and had the following loadings per variable: TDS = 0.584, salinity = 0.581, conductivity = 0.561 and pH = − 0.083, indicating that pH has little influence compared to the other water parameters. The PCA graphs are shown in Additional file 2: Figure S4.

**Fig. 5:**
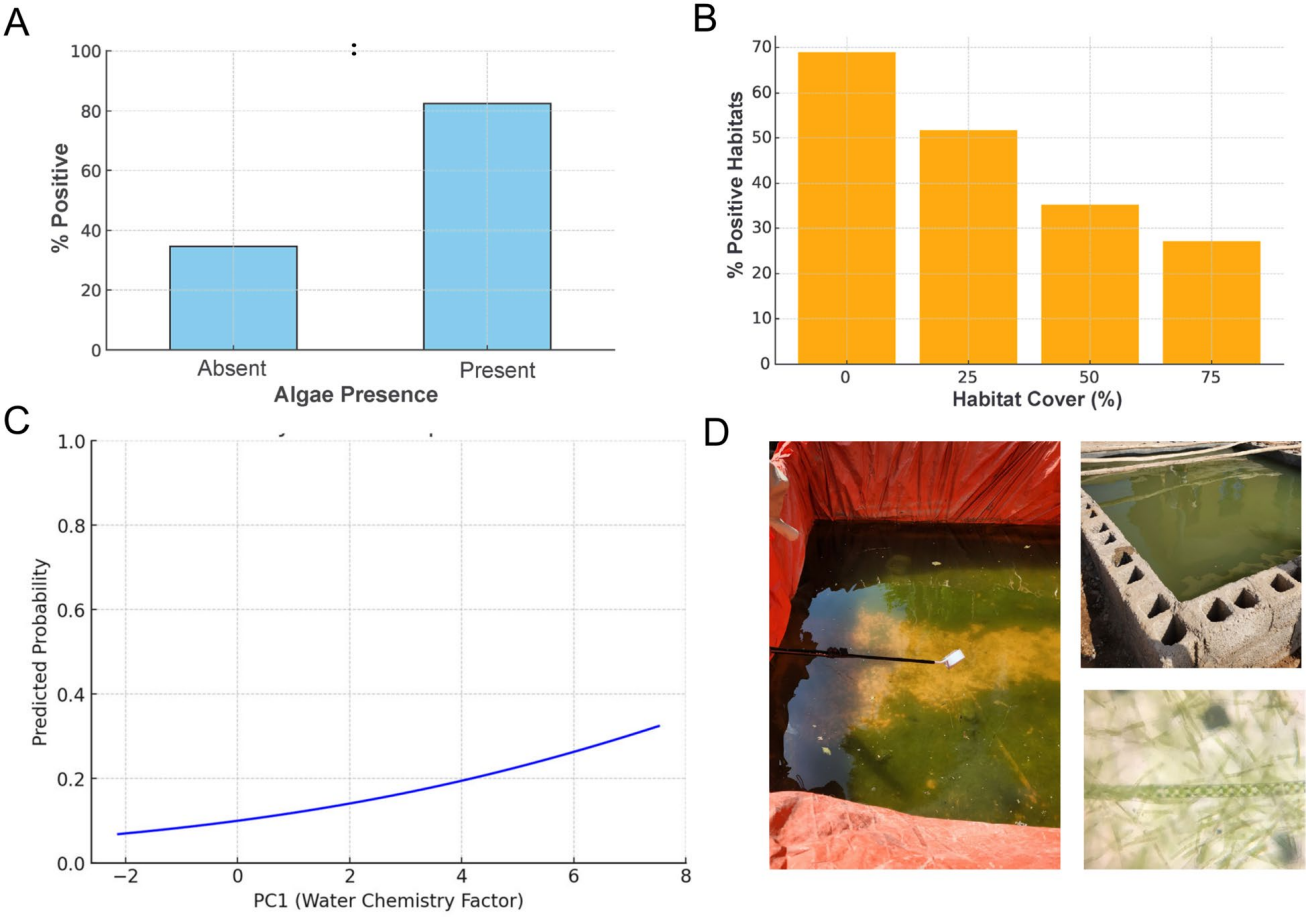
Factors associated with *Anopheles stephensi* positivity. **A** Association between algae and positivity, **B** association between cover and positivity, **C** association between PC1 (water quality index) and positivity. **D** Examples of algae-positive habitats found infested with *An. stephensi* and microscope magnification (400×) showing the structure of the green filamentous algal aggregates. PC1, Principal component 1

*Anopheles stephensi* positivity was significantly associated with the presence of green filamentous algal aggregates (OR 6.00, 95%CI 2.76–13.04), followed by water chemistry (OR 1.21, 95% CI 1.03–1.42) and habitat cover (OR 0.98, 95% CI 0.96–0.98) (Table 2). Water turbidity was not associated with positivity (Table 2). The model also captured significant differences between habitat types (plastic drums and tanks being less positive than residential cisterns), with Jigjiga having less positivity than the other towns (Table 2). Figure 5 shows the relationship between *An. stephensi* positivity and the significant variables obtained from the model. More than 80% of habitats with visual confirmation of green filamentous algal aggregates were positive for *An. stephensi*, compared to only 34.6% when algae were absent (Fig. 5a). As for habitat cover, a linear reduction in positivity occurred with a linear increase in habitat cover, with 69% of habitats that were uncovered found to be positive for *An. stephensi* (Fig. 5b). Water chemistry only explained a small fraction of all positive habitats, but *An. stephensi* positivity increased with PC1 (Fig. 5c).

**Table 2:** Factors associated with *Anopheles stephensi* positivity and productivity in the three top habitats in the three towns located in Eastern Ethiopia

Metric	Predictor^a^	Odds ratio or incidence rate ratio	95% CI	*P*-value
Habitat positivity	Cover (per 1% increase)	0.987	0.96–0.98	0.0025*
	Green filamentous algal aggregates	6.00	2.76–13.04	< 0.001*
	Habitat B vs A	0.87	0.40–1.94	0.728
	Habitat C vs A	0.29	0.16–0.53	< 0.001*
	Turbidity Medium vs Clean	1.86	0.93–3.92	0.082
	Turbidity Turbid vs Clean	1.10	0.45–2.53	0.815
	Town Logiya vs Jigjiga	4.77	2.52-9.00	< 0.001*
	Town Semera vs Jigjiga	5.57	3.00–10.37	< 0.001*
	PC1 (Water Chemistry)	1.21	1.03–1.42	0.018
Habitat productivity	Cover (per 1% increase)	0.98	0.97–0.99	0.001*
	Green filamentous algal aggregates	2.56	1.13–5.76	0.024*
	Habitat B vs A	0.81	0.38–1.70	0.579
	Habitat C vs A	0.31	0.15–0.64	0.001*
	Turbidity Medium vs Clean	1.10	0.52–2.32	0.810
	Turbidity Turbid vs Clean	1.63	0.63–4.25	0.315
	Town Logiya vs Jigjiga	16.62	8.25–33.51	< 0.001*
	Town Semera vs Jigjiga	11.79	5.71–24.36	< 0.001*
	PC1 (water chemistry factor)	1.13	0.94–1.31	0.190

*CI* Confidence interval,* PC1* principal component 1

*Significant association with *Anopheles stephensi* positivity and productivity

^a^Three top habitats in terms of *Anopheles stephensi* positivity and productivity: A = residential cisterns, B = construction pits, C = ground-level tanks

**Fig. 6:**
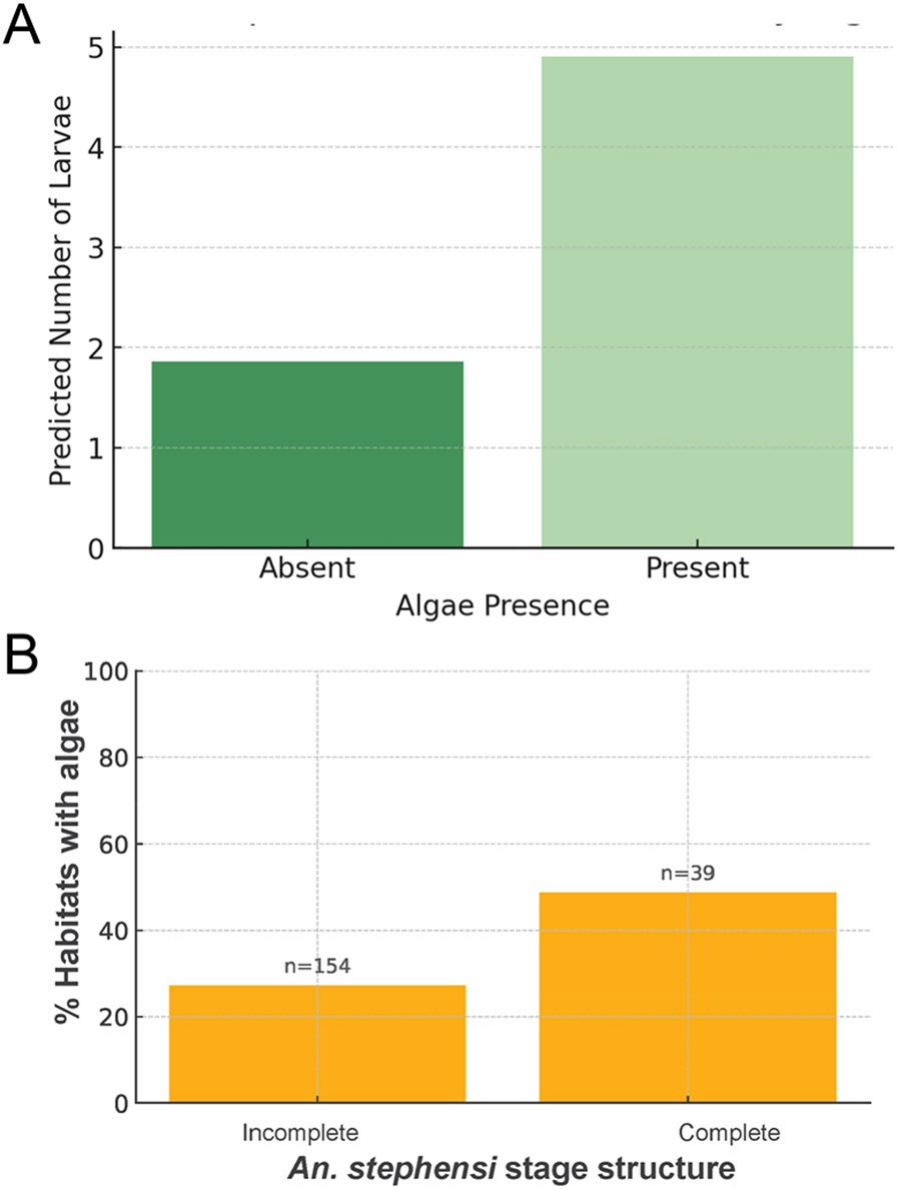
*Anopheles stephensi* productivity and green algal aggregate presence. **A** Predicted number of larvae per habitat from best-fitting negative binomial model (Table 2). **B** Percentage of habitats with green filamentous algal aggregates in which the *An. stephensi* stage structure was found to be complete (all larval stages and pupae), compared to incomplete (at least one stage missing)

Productivity, measured as number of larvae and pupae per habitat, was also primarily explained by the presence of green filamentous algal aggregates (incidence risk ratio [IRR] 2.56, 95% CI 1.13–5.76), followed by the percentage cover of each habitat (IRR 0.98, 95% CI 0.97–0.99) (Table 2). Water chemistry and turbidity did not have a significant association with productivity (Table 2). Figure 6 shows the predicted number of *An. stephensi* when green filamentous algal aggregates were present. Another way of looking at the role of filamentous algal aggregates on productivity was exemplified by the significant relationship found between the finding of a complete stage structure in a habitat and the presence of algae (*χ*^2^ = 5.66,* P* = 0.0173; Fig. 6b).

## Discussion

In eastern Ethiopia, where most *An. stephensi* populations are currently found [13, 14], the dry season can extend for up to 3–4 months and often be erratic. By conducting simultaneous systematic dry season collections across three urban centers from two regions of Ethiopia, we have obtained new knowledge on the drivers of *An. stephensi* positivity and productivity within a critical time period for population persistence. Among all possible habitat types found in the three towns, *An. stephensi* was predominantly found in residential cisterns, construction pits and ground-level tanks. Construction pits were the most productive, validating findings from a previous survey conducted in Jigjiga [9]. Interestingly, in the most infested and productive habitats (residential cisterns, construction pits, ground-level tanks), the presence of green filamentous algal aggregates was the most important factor associated with *An. stephensi* positivity or productivity, followed by the lack of any habitat cover. While water chemistry played a role in explaining habitat positivity, the lack of statistical association between habitat positivity and habitat productivity may be indicative of a differential influence of water conditions on oviposition site selection and larval success.

*Anopheles stephensi* is found across diverse regions of Ethiopia, but the results of the present study show that both positivity and productivity can vary markedly across these locations. While similar types of habitats were found across regions, we acknowledge that elevation and temperature differences may have explained why Jigjiga was associated with a lower productivity than Semera and Logiya, but also with more habitats with a complete stage structure (indicative of a more “productive” environment). Jigjiga is found at 1634 m a.s.l., and even during the hot dry season water temperatures averaged 24.4 °C. Such conditions, while possibly delaying larval development and explaining low overall densities, are less extreme than in those in Semera and Logiya, likely leading to more stable population structures and complete larval stage distributions. Semera and Logiya are not only found further north but also at a much lower elevation (430 m a.s.l.), and water temperatures reached an average of 29 °C. Interestingly, the maximum temperature of the water in which *An. stephensi* was found in Semera and Logiya (35.7 °C and 34.2 °C, respectively) appeared to be around the peak value of the estimated thermal response of *An. stephensi* [23], implying that such localities may be highly suitable for the establishment and potential transmission of malaria. A recent observational study of malaria trends identified increases in urban malaria transmission in Semera, particularly after 2018 when *An. stephensi* was detected in the town [12]. Despite the well-established presence of *An. stephensi* in Jigjiga since 2018 [9, 13–15], no urban malaria cases have been reported to the regional board of health in that town. As our study expands, focusing in areas of Jigjiga with the most potential for human–*An. stephensi* contacts may provide further insight on the possibility of localized malaria transmission even in areas that historically are malaria-free. Taken together, these findings suggest that the context of *An. stephensi* invasion will also have to be considered when assessing malaria transmission risk in urban settings of eastern Ethiopia.

In Eastern Ethiopia, a wide array of potential *An. stephensi* larval habitats are generally found in and around houses [9, 13, 24]. In the three study towns, most small containers and tires were empty during the dry season, and even when they contained water, their positivity and productivity were relatively low. Our study also detected a very small number of water puddles (consequence of failing water infrastructure) and drainage areas from car wash businesses that tested positive for *An. stephensi*. While the number of* An. stephensi* larvae was very small to justify their role in the seasonal persistence of *An. stephensi* in the dry season, our finding provides insight into the potential for such understudied habitat types to be more relevant in terms of productivity during the rainy season or in other towns where *An. stephensi* is found. This plasticity in habitat exploitation was also reported from Kenya, where *An. stephensi* was found infesting artificial containers in urban areas and a also variety of artificial containers, puddles and surface water from rural areas where they often co-occurred with *Anopheles arabiensis* [25].

*Anopheles stephensi *positivity and productivity were disproportionately high in a subset of habitats, particularly large-volume containers, such as residential cisterns, construction pits and ground-level tanks. Among the three main habitats, construction pits were predicted to produce the most adults across the three towns, confirming previous findings from a systematic review [16] and prior data from Jigjiga [9]. Similarly, in India, large water reservoirs and elevated water tanks are primary habitats of *An. stephensi*, contributing to urban malaria transmission throughout the rainy and dry seasons [26–28]. Interestingly, in one of these studies [26], the majority of larvae from habitats other than elevated tanks originated from a construction pit, which contributed to > 90% of all immatures. The permanence of water and abundant sun exposure, organic matter and algae generate conditions in such habitats that favor oviposition and larval development of *An. stephensi*. The importance of construction sites for *An. stephensi* productivity and malaria transmission has been highlighted in the Indian context [29]. Migrant and permanent workers in Indian construction industries generally have a poor understanding of malaria transmission [30] and often experience malaria [31]. Previous findings from Jigjiga, together with the results of our current study, highlight the role of construction sites as an environment favoring *An. stephensi* persistence and productivity [9]. Given our findings and the experience in India, a broader approach to construction sites in eastern Ethiopia, which is currently experiencing a large urban expansion and construction boom [9], may be required. A focus on better quantifying malaria exposure among construction workers, improving current understanding of the role of migrant workers from malaria endemic areas as sources of *Plasmodium* introduction into cities and exploring regulatory and political approaches for reducing *An. stephensi* productivity may help fill key knowledge gaps needed to generate integrated approaches for vector control and disease management.

Our study centrally focused on estimating measures of *An. stephensi* productivity, such as larval indices and the stage structure of populations within habitats. We found that 80% of larvae were collected in 19% of the habitats, an overwhelming majority of which corresponded to residential cisterns and construction pits, confirming previous finding of a 77% predominance in such habitats [18]. Among all sites, construction pits disproportionately contributed the most larvae and, for Semera and Jigjiga, the most pupae. During our study period, construction pits were very common in Semera and Jigjiga. Both towns are currently experiencing rapid urbanization, fueled by different drivers. While Jigjiga’s growth is driven by political stability, safety and external investment [9], Semera has become one of Ethiopia’s newest capitals after the regional government decided in 2007 to move the Afar Region’s capital from Asaita to Semera. Furthermore, the town sits on one of the busiest trade corridors of Ethiopia, connecting Djibouti with Addis Ababa. Given this high demand for construction and limited water sources for mosquitoes during the dry season, the number of construction sites in both towns has provided ample habitats for *An. stephensi* to thrive. The large demand for construction and high connectivity to shipping ports may be two of the factors explaining the high *An. stephensi* genetic diversity in Semera and Jigjiga compared to other towns in Ethiopia [32].

Our study provided a second measure of productivity, centered on the stage structure of *An. stephensi* across larval habitats. Linking stage structure information with a Lefkovitch matrix model, we quantified simple projections of the potential contribution of each habitat site to the adult mosquito population. In the dry season, the most productive habitats that also contained a complete stage structure (indicative of multiple oviposition events) were residential cisterns, construction pits and ground-level tanks. Construction sites were predicted to produce the most adults. Focusing on the stage distribution of immatures provides a unique perspective on the potential contribution of certain habitat types. For most small containers and surface water, an incomplete stage structure was indicative of infrequent oviposition and low contribution to the population. This was further emphasized by the low predicted adult emergence from such habitats. For the top three habitats, however, a complete stage structure provided strong evidence of their role as sources of emerging adults and important oviposition sites. Knowledge of *Anopheles* spp. productivity is scarce (e.g. [33]), but it is important to define LSM targets when productive habitats are few, fixed and findable [19]. Our study also provides valuable information for rapid population sampling of *An. stephensi*. In areas where there is confirmed presence of the vector; quantifying the presence of each instar (young, old, pupae) may provide a rapid approach for identifying key habitats that may be the target of LSM. As more evidence of *An. stephensi* is generated, developing guidelines for standardized sampling can benefit from the understanding of mosquito population structure and productivity, as has been done for *Ae. aegypti* [34].

In the present study, *An. stephensi* positivity was explained by a set of interrelated factors. The presence of green filamentous algal aggregates, alone explained > 80% of positivity and was the most important factor explaining productivity, followed by the degree of cover of each habitat and an index of water quality. The visual presence of algae (Fig. 5d) clearly showed a measure of habitat quality that is easy to determine in the field and which should be recorded in future studies of *An. stephensi*. The presence of algae has been identified as an important factor for other *Anopheles* species [35, 36], and experimental studies show that algae are an important component in larval diet [37] and may play a role as oviposition cue [38]. In India, the visual presence of algae was positively associated with *An. stephensi* presence and productivity [26]. Uncovered habitats not only had more *An. stephensi* but also were more productive and contained more green filamentous algal aggregates, which is a consequence of higher sun exposure. The presence of algae was also more common in habitats with water that was less frequently used for residential consumption (construction pits and abandoned cisterns). Among all water chemical properties, an index composed of conductivity, pH, TDS and salinity was significant in explaining *An. stephensi* positivity. As increased evaporation leads to higher ion concentrations, water quality and algae presence may be influenced by the source and frequency of habitat refill. Consequently, an understanding of water use practices becomes relevant for understanding *An. stephensi* larval ecology and productivity.

While our study provides unprecedented details, we also acknowledge several limitations. Although our study focused on larval productivity and positivity, we failed to detect enough adult *An. stephensi* to correlate such measures to the potential for human exposure. The poor detection of adult *An. stephensi* is a challenge for most field research and requires further study [16, 25, 39]. We selected three towns that differed in climate, culture and environmental conditions to capture sufficient variability to arrive to emerging patterns. We also acknowledge that this study was conducted during a single dry season and that different patterns may emerge as more towns or annual variability data are considered in future research. We used a visual measure of turbidity, rather than a sensor, which may have led to inaccurate estimates. Costs prevented us from using multiple devices, but future research will focus on this water quality measure in a more quantitative way. We acknowledge that other towns may show other properties or less common habitats in the dry season and, as such, future work replicating our study design should be pursued to validate our findings. While we visually confirmed the presence of algae (Fig. 5d), the species or genus of algae associated with *An. stephensi* remains to be confirmed. A topic of future research will be the study of algae and *An. stephensi*, including which algal species are associated with productivity.

Taken together, our findings further increase support for considering the dry season in Ethiopia as an important period for *An. stephensi* population persistence and for LSM deployment [18]. During this crucial population bottleneck, fewer habitat types are productive. Among such habitats, residential cisterns, construction pits and ground-level tanks, given their large size, are easily findable within a community. Multiple options can be considered for LSM of *An. stephensi* within such habitats. Our results imply that covering habitats with the aim to provide shade as protection from solar irradiation may significantly reduce *An. stephensi* positivity and productivity. In the dry season, evaporation is a major challenge faced by water scarce communities. As a consequence, communities use metal sheeting to cover cisterns. Developing a cost-effective method for habitat cover may provide multiple gains beyond mosquito control and should be further explored, as has been done in the Indian context [27]. In construction pits and habitats with algae, larvivorous fish [40] may play an important role in reducing mosquito populations and controlling *An. stephensi*. Finally, using larvicides that are rationally applied in key habitats during the dry season may lead to *An. stephensi* population control [18], particularly if they are formulated to be long-lasting. As more knowledge on *An. stephensi* habitat use emerges, the need for evaluating such methods (in isolation or combined) becomes a major public health priority to reduce the threat of mosquito expansion and to support targeted, cost-effective, larval control strategies during the dry season in urban Ethiopia.

## Conclusions

During the prolonged and often erratic dry season experienced by three eastern Ethiopia towns infested with *An. stephensi*, residential cisterns, construction pits and ground-level tanks emerged as the dominant *An. stephensi* habitats, with construction pits confirmed as the most productive of these sites. Across these key habitats, the presence of green filamentous algal aggregates was the primary factor associated with larval positivity and productivity, followed by the absence of physical cover (lack of shade). These findings highlight the importance of specific urban habitat types and ecological drivers in sustaining *An. stephensi* populations through the dry season, with direct implications for targeted larval source management strategies.

## Supplementary Information

**Additional file 1:** Dataset.

**Additional file 2: Figure S1.**Map of study sites.**Figure S2.**Photo of representative sites for each habitat category.**Figure S3.**Correlation matrix of water quality indices.**Figure S4.**PCA graphs.**Table S1.**Model results of association ofAn. stephensiwith habitat type.**Table S2.**Model results of association ofAn. stephensiand city.**Table S3.**Empirical stage distribution of An. stephensi.

## Data Availability

The data used to run all analyses is included with this manuscript as Additional file 1: Dataset S1.

## Abbreviations

GLMM: Generalized linear mixed models
GPS: Global Positioning System
IRR: Incidence risk ratio
LSM: Larval source management
PCA: Principal component analysis
TDS: Total dissolved solids
