# Supplementary material for: Habitat heterogeneity and green filamentous algae influence the larval ecology of Anopheles stephensi during the dry season in Eastern Ethiopia
Source: Parasit Vectors. 2025 Nov 14;18:461. doi: 10.1186/s13071-025-07100-7 (PMC12619324; doi:10.1186/s13071-025-07100-7)
Supplement: Supplementary file 2 — Additional file 2: Figure S1.Map of study sites.Figure S2.Photo of representative sites for each habitat category.Figure S3.Correlation matrix of water quality indices.Figure S4.PCA graphs.Table S1.Model results of association ofAn. stephensiwith habitat type.Table S2.Model results of association ofAn. stephensiand city.Table S3.Empirical stage distribution of An. stephensi. [file 13071_2025_7100_MOESM2_ESM.docx]

**Algae and habitat heterogeneity influence the larval ecology of *Anopheles stephensi* in Ethiopia**

**Supplemental Figures and Tables**

**Table S1.** Results of a binomial GLMM comparing habitat positivity across all habitat types in the three study sites. Baseline habitat was residential cisterns [A], whereas other habitats (by letter) were: B. Construction pit. C. Ground level tanks. D. Tires. E. Small plastic containers. F. Bottles. G. Puddles from rain or burst pipes. H. Car wash drainage. I. Surface water.

| **Habitat Type** | **Odds Ratio** | **CI**  **2.5%** | **CI 97.5%** | **P-value** |
| --- | --- | --- | --- | --- |
| Intercept | 0.92 | 0.62 | 1.37 | 0.683 |
| Habitat: B | 2.40 | 1.38 | 4.18 | **0.002** |
| Habitat: C | 0.39 | 0.24 | 0.64 | **<0.001** |
| Habitat: D | 0.54 | 0.13 | 2.30 | 0.407414 |
| Habitat: E | 0.14 | 0.04 | 0.41 | **<0.001** |
| Habitat: G | 2.02 | 0.74 | 5.50 | 0.16958 |
| * Habitats H and I had few observations and the model did not provide OR estimates | | | | |

**Table S2.** Productivity across the three cities. Result from a generalized linear model comparing the sum of immature *An. stephensi* per habitat (top) or the number of immature *An. stephensi* per dip (bottom) across study sites. Jigjiga was set as the baseline for all models.

| **Metric** | **Variable** | **IRR** | **CI 2.5%** | **CI 97.5%** | **P-value** |
| --- | --- | --- | --- | --- | --- |
| **Sum *An. stephensi* per habitat** | Intercept | 2.87 | 2.49 | 3.31 | **<0.001** |
|  | City: Logiya | 11.29 | 9.06 | 14.06 | **<0.001** |
|  | City: Semera | 8.94 | 7.12 | 11.22 | **<0.001** |
|  |  |  |  |  |  |
| **No. *An. stephensi* per dip** | Intercept | 0.14 | 0.10 | 0.20 | **<0.001** |
|  | City: Logiya | 17.57 | 11.81 | 26.13 | **<0.001** |
|  | City: Semera | 9.65 | 6.38 | 14.60 | **<0.001** |

**Table S3.** Empirical *An. stephensi* stage distribution by habitat and study site

|  |  | Larvae | |  |
| --- | --- | --- | --- | --- |
| City | Habitat | Young (I-II) | Old (III-IV) | Pupae |
| Jigjiga | A | 45 | 15 | 16 |
| Jigjiga | B | 265 | 153 | 99 |
| Jigjiga | C | 165 | 79 | 51 |
| Jigjiga | D | 1 | 0 | 0 |
| Jigjiga | E | 0 | 2 | 0 |
| Jigjiga | G | 0 | 8 | 1 |
| Jigjiga | H | 0 | 0 | 0 |
| Logiya | A | 317 | 11 | 30 |
| Logiya | B | 1973 | 30 | 49 |
| Logiya | C | 1399 | 5 | 105 |
| Logiya | D | 88 | 0 | 19 |
| Logiya | E | 4 | 0 | 0 |
| Logiya | G | 702 | 45 | 41 |
| Logiya | H | 0 | 0 | 0 |
| Semera | A | 1228 | 23 | 45 |
| Semera | B | 1712 | 34 | 112 |
| Semera | C | 242 | 0 | 12 |
| Semera | D | 0 | 0 | 0 |
| Semera | E | 4 | 0 | 0 |
| Semera | G | 15 | 0 | 0 |
| Semera | I | 1 | 0 | 10 |

**Figure S1.** Distribution of sampling sectors and clusters by study site.


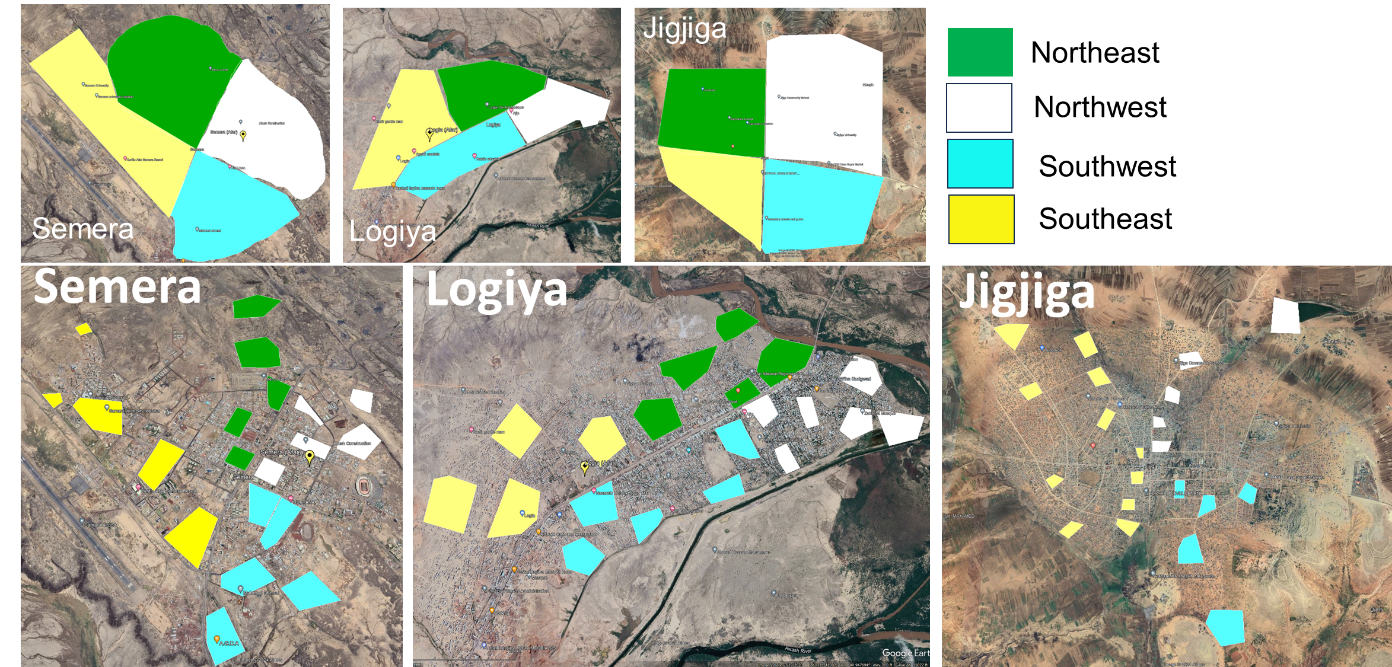


**Figure S2. Photos describing each of the 8 categories of habitats found in the study areas.**


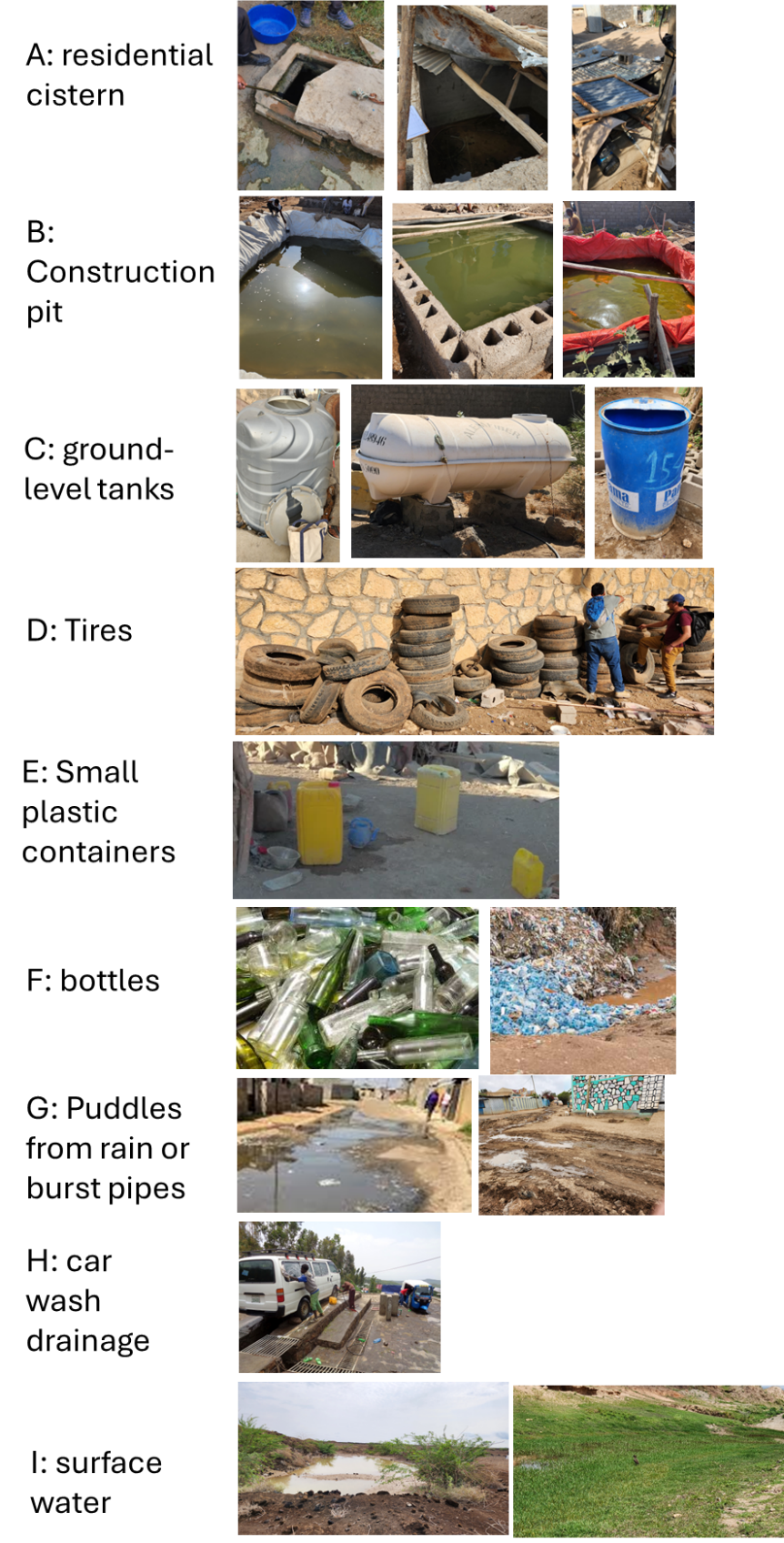


**Figure S3.** Correlation matrix of all water quality indices considered in the multivariate analysis of factors associated with *An. stephensi* positivity and productivity in three cities of Ethiopia.


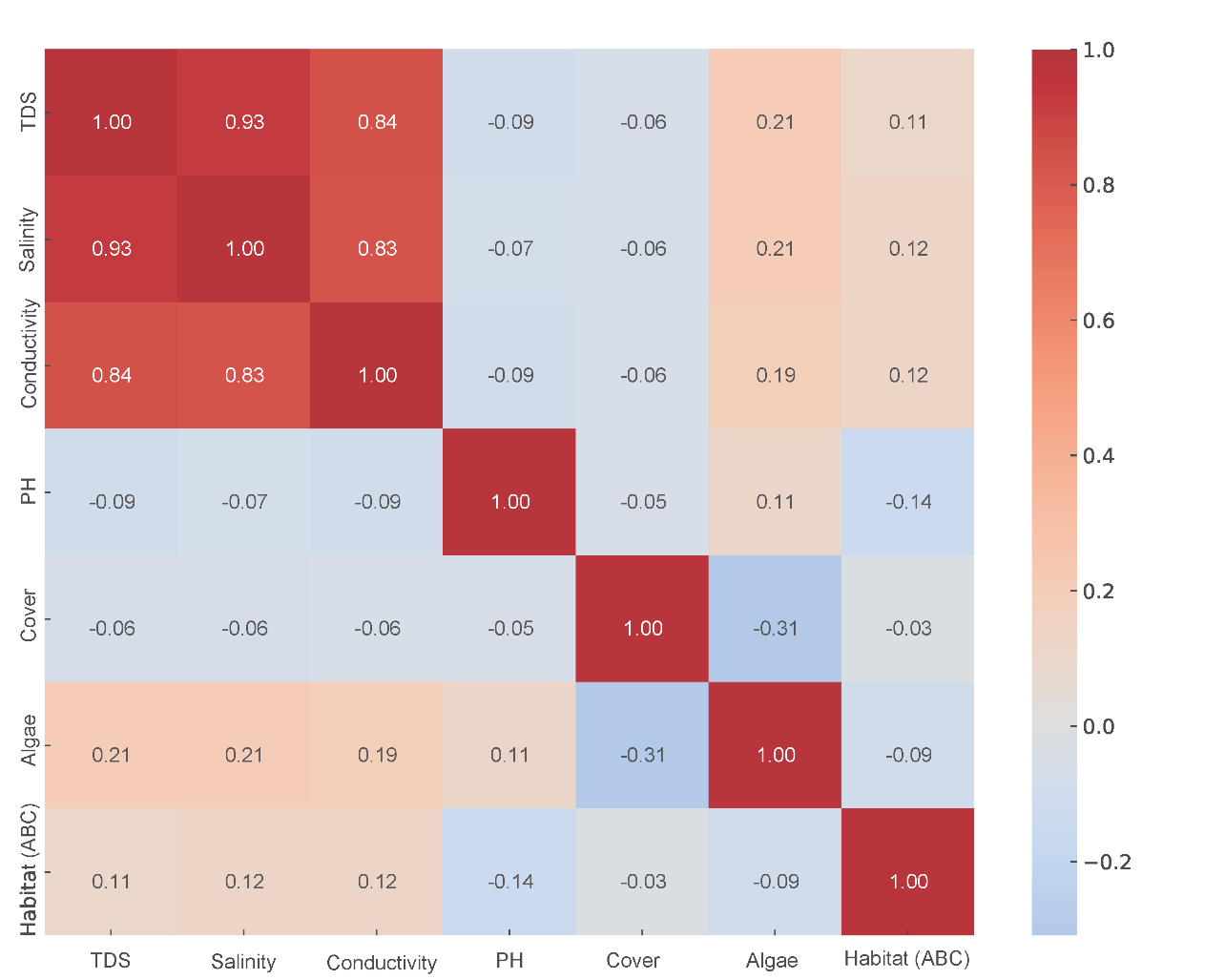


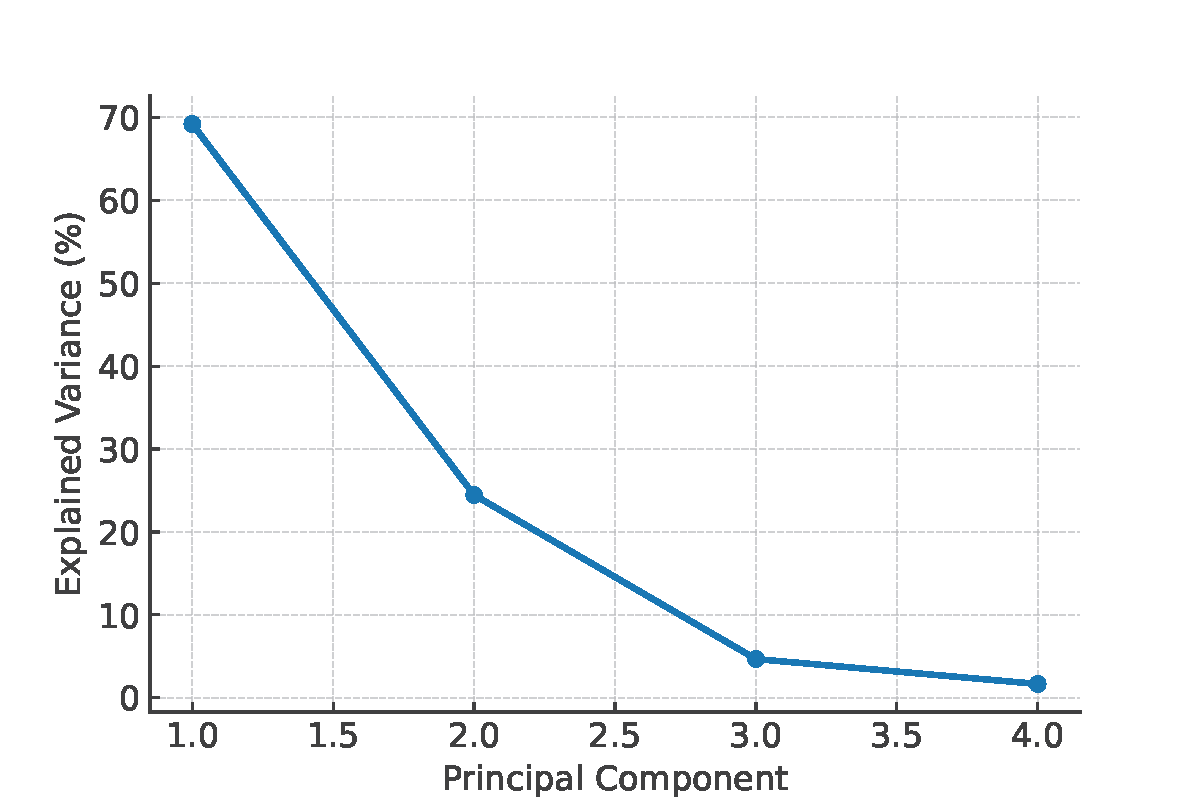
Figure S4. Results from the PCA conducted on water chemistry parameters to identify PC1, the parameter used in analyses. (A) Loadings of each PCA, expressed as percentage of variance explained. (B) Biplot of PCA1 and PCA2.

(A)


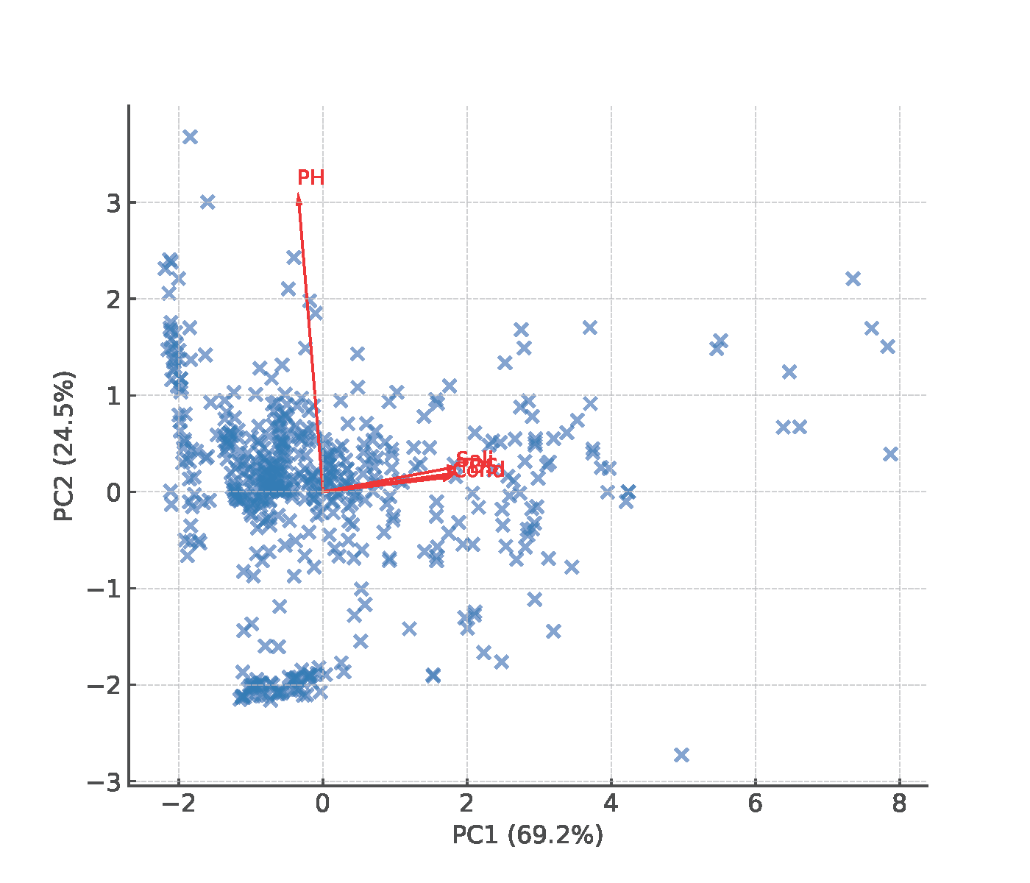


(B)
